# Beta-Blockers and Their Current Role in Maternal and Neonatal Health: A Narrative Review of the Literature

**DOI:** 10.7759/cureus.44043

**Published:** 2023-08-24

**Authors:** Andrea Martinez, Mohit Lakkimsetti, Sameep Maharjan, Muhammad Ammar Aslam, Anouksha Basnyat, Shashwat Kafley, Subrahmanya saketh Reddy, Saima S Ahmed, Waleed Razzaq, Susmitha Adusumilli, Uzzam Ahmed Khawaja

**Affiliations:** 1 Medical School, Universidad Autonoma de Guadalajara, Zapopan, MEX; 2 Internal Medicine, Mamata Medical College, Khammam, IND; 3 General Practice, Patan Academy of Health Sciences, Kathmandu, NPL; 4 Medical School, Sargodha Medical College, University of Health Sciences, Sargodha, PAK; 5 General Practice, Hospital for Advanced Medicine & Surgery (HAMS), Kathmandu, NPL; 6 Medical School, Enam Medical College and Hospital, Dhaka, BGD; 7 Internal Medicine, Area Hospital Patancheru, Hyderabad, IND; 8 Vascular Surgery, Dow International Medical College, Karachi, PAK; 9 Internal Medicine, Services Hospital Lahore, Lahore, PAK; 10 Medical School, Chongqing Medical University, Chongqing, CHN; 11 Paediatrics and Child Health, Aga Khan University Hospital, Karachi, PAK

**Keywords:** future directions, lactation, neonates, maternal and fetal health, hypertension, cardiovascular disease, pregnancy, beta-blockers

## Abstract

Beta-blockers are a class of medications that act on beta-adrenergic receptors and are categorized as cardio-selective and non-selective. They are principally used to treat cardiovascular conditions such as hypertension and arrhythmias. Beta-blockers have also been used to treat non-cardiogenic indications in non-pregnant individuals and the pediatric population. In pregnancy, labetalol is the mainstay treatment for hypertension and other cardiovascular indications. However, contraindications to certain sub-types of beta-blockers include bradycardia, heart failure, obstructive lung diseases, and hemodynamic instability. There is conflicting evidence of the adverse effects on fetal and neonatal health due to a scarce safety and efficacy profile, and further studies are necessary to understand the pharmacokinetics of the different classes of beta-blockers in pregnancy and fetal health. Understanding the hemodynamic changes during the stages of pregnancy is important to target a more beneficial therapy for both mother and fetus as well as better neonatal outcomes. Beta-blocker use in the pediatric population is less documented in studies but does have the potential to treat various cardiogenic and non-cardiogenic conditions. Future comprehensive studies would further benefit the direction of beta-blocker treatment during pregnancy in neonates and pediatrics.

## Introduction and background

Beta-blockers are primarily used to treat cardiovascular diseases and other associated conditions [1]. These are the molecules that compete with catecholamines for the binding site on beta-adrenergic receptors. There are currently more than 20 antagonists that are commercially available for clinical use. The action of beta-blockers in the cardiovascular system includes negative inotropic and bradycardic effects, which translate into a lower cardiac output. In addition, antagonism of beta-1 receptors in the juxtaglomerular cells can reduce the activity of the renin-angiotensin system, resulting in decreased blood pressure [2-9]. Beta-blockers work on three different types of beta receptors. Beta-1 receptors are primarily located in the heart and mediate cardiac activity. Beta-2 receptors, with their diverse locations in many organ systems, are responsible for controlling metabolic activity and smooth muscle relaxation. Beta-3 receptors are responsible for lipolysis and are clinically less relevant [1].

Types of beta-blockers

Beta-blockers are classified as either non-selective or selective. Non-selective agents bind to both beta-1 and beta-2 receptors and induce antagonizing effects via both receptors. Examples include propranolol, carvedilol, sotalol, and labetalol. Selective beta-blockers act on specific receptors. Beta-1 receptor-selective blockers like atenolol, bisoprolol, metoprolol, and esmolol only bind to the beta-1 receptors; therefore, they are cardio-selective [10-12]. Further examples of cardioselective ones include atenolol, betaxolol, bisoprolol, esmolol, acebutolol, metoprolol, and nebivolol [13]. The FDA-approved uses of beta-1 selective blockers include hypertension, chronic stable angina, heart failure, post-myocardial infarction, and decreased left ventricular function after a recent myocardial infarction [14].

Mechanism of Action

Different hormones work on different beta receptors. Catecholamines, epinephrine, and norepinephrine bind to beta-1 receptors and increase cardiac automaticity as well as conduction velocity. Beta-1 receptors also induce renin release at the level of the kidneys. In contrast, binding to beta-2 receptors causes relaxation of the smooth muscles along with increased metabolic effects such as glycogenolysis. Once the beta-blockers bind to the beta-1 and beta-2 receptors, they inhibit these effects, e.g., the chronotropic and inotropic effects on the heart undergo inhibition, and the heart rate slows down as a result of the beta-blocker attack. Beta-blockers also decrease blood pressure via several mechanisms, including decreased renin and reduced cardiac output. The negative chronotropic and inotropic effects lead to decreased oxygen demand and help relieve angina symptoms. These medications also prolong the atrial refractory periods and have a potent antiarrhythmic effect [13,14].

Despite the common mechanism of many beta blockers, there are several differences in their specific activities. The most important pharmacodynamic difference among beta-blockers is their selectivity for adrenergic receptors and their subtypes. On the basis of this selectivity, they are classified into three generations. Representatives of the first generation are nonselective antagonists of receptors of types beta-1 and beta-2. Representatives of the second generation have selectivity for beta-1 receptors compared to beta-2, also called cardioselective beta blockers, but this feature is dose-dependent. Third-generation beta blockers are also known as vasodilators as they block α1-adrenoreceptors and activate beta-3 receptors, which increase nitric oxide synthase (NOS) activity and nitric oxide generation. An understanding of the differences among the generations of beta-blockers is important to understand their utilization [9].

Beta-Adrenergic Antagonists

Beta-receptor antagonists block the effects of catecholamines like epinephrine and norepinephrine on beta-adrenoceptors. Beta-blocking drugs function by binding to beta-receptors, reducing the availability of these receptors to catecholamines like epinephrine or norepinephrine and other beta-agonist drugs through competitive inhibition [15,16].

Physiological Mechanism of Beta-Adrenoceptors

Beta-adrenoceptors consist of three subtypes, namely, beta-1, beta-2, and beta-3, all of which belong to the G protein-coupled receptor (GPCR) family. In the heart, beta-1 receptors are abundant compared to beta-2 receptors, present in a molar ratio of 4:1. Beta-1 receptors activate a stimulatory G protein (Gs), whereas beta-2 receptors activate both a stimulatory G protein (Gs) and an inhibitory G protein (Gi), with a predominant activation of Gs. On the other hand, beta-3 receptors are coupled to the inhibitory Gi protein, which also leads to additional intracellular signaling such as activation of NOS, activation of guanylate cyclase (GC), and formation of cyclic guanosine monophosphate (cGMP) [1,10].

Beta-adrenoceptors have a dual impact on the cardiovascular system. They stimulate the heart to contract more forcefully and increase cardiac output, and they also promote the release of renin from the juxtaglomerular cells of the kidney, indirectly contributing to blood pressure regulation. Furthermore, activation of beta-1 adrenergic receptors encourages the secretion of renin, which enhances the activity of the renin-angiotensin system (RAS), a significant contributor to the development of various cardiovascular diseases [1,10,16].

Classification of beta-adrenergic antagonists

First-Generation Beta-Blockers (Non-selective)

First-generation beta-blockers, also known as non-selective beta-blockers, function by competitively blocking both beta-1- and beta-2-adrenergic receptors. A common example of a non-selective beta-blocker is propranolol, timolol, nadolol, etc. [1,15,16]. First-generation beta-blockers lower both systolic and diastolic blood pressure by decreasing the heart's contractile strength and rate, which reduces cardiac output as well as the renin-angiotensin-aldosterone system (RAAS) activity. Therefore, they are used to treat hypertension, angina, arrhythmias, coronary artery disease, and post-myocardial infarction and have anti-ischemic effects. They also increase peripheral vascular resistance through antagonistic activity on beta-2 receptors in the peripheral vasculature. Timolol is used topically for glaucoma [1,15]. However, propranolol is contraindicated in patients with diabetes due to the occurrence of antagonistic adverse effects like hypoglycemia and in lung pathologies like asthma or chronic obstructive pulmonary disease due to bronchospasm [1].

Second-Generation Beta-Blockers (Selective)

Second-generation beta-blockers, also known as cardioselective beta-blockers, selectively block beta-1 adrenergic receptors, i.e., metoprolol, atenolol, betaxolol, and nebivolol. They have a higher affinity for beta-1 receptors compared to beta-2 receptors [1,15,16]. These medications are frequently prescribed for various cardiovascular conditions, such as hypertension, angina, and congestive heart failure [11]. Beta-1 selective beta-blockers work by reducing the heart rate and contractility of the heart, which results in a decrease in cardiac output and blood pressure. This mechanism of action is particularly beneficial for patients with hypertension, as it helps to decrease the workload on the heart and improve overall cardiac function [1,15,17]. Compared to non-selective beta-blockers, beta-1 selective beta-blockers have a lower risk of adverse effects such as bronchospasm and hypoglycemia in patients with asthma or diabetes, respectively [1,15].

Third-Generation Beta-Blockers

Third-generation beta-blockers, including nebivolol, labetalol, and carvedilol, have been developed to target not only beta-1 and beta-2 receptors but also beta-3 receptors. Nebivolol is a nitric oxide-dependent vasodilator that widens blood vessels and improves endothelial function. This makes it useful for treating high blood pressure and heart failure with preserved ejection fraction [1,18]. Carvedilol, as well as labetalol, has alpha-blocking properties and has been shown to reduce peripheral vascular resistance and improve left ventricular function in heart failure patients. However, both medications have adverse effects such as headache, fatigue, dizziness, bradycardia, hypotension, and an increased risk of bronchospasm in asthmatics. Despite this, third-generation beta-blockers offer unique mechanisms of action and clinical benefits for the treatment of cardiovascular diseases [1,19].

Indications of beta-blockers

Beta-blockers have demonstrated long-term beneficial effects on mortality and cardiovascular disease (CVD) when used in patients with heart failure or acute myocardial infarction [20]. In this section, we comprehensively shed light on the cardiogenic and non-cardiovascular indications of beta-blockers.

Cardiovascular Indications of Beta-Blockers

Heart failure (HF) is a heterogeneous clinical syndrome arising from cardiac overload and injury that leads to considerable morbidity and mortality [21]. With time, there is an increase in adrenergic tone and neurohormonal activity that leads to progressive left ventricular (LV) dysfunction and structural remodeling, which is marked by a declining LV ejection fraction as well as dilation and hypertrophy [22]. Persistent activation of the sympathetic nervous system in patients with heart failure with reduced ejection fraction is evidenced by elevated plasma levels of epinephrine and norepinephrine [23]. The increased levels of catecholamine release lead to chronic and persistent stimulation of the beta receptors of the heart, which gives rise to the resulting dysfunction and harmful consequences of a failing heart [24,25]. The failing heart shows a decreased beta receptor-mediated stimulation of adenylate cyclase stimulation and contractile response [26]. This decrease in responsiveness is related to changes in the expression of the receptor and function [27]. This deleterious effect of sympathetic nervous system stimulation is counteracted by beta-blockers [28].

Ischemic heart disease is a major cause of death and disability globally, and angina presents as its most common symptom [29]. Atherosclerosis of the coronary arteries has been a widely accepted cause of angina pectoris for over two hundred years. Similarly, the spasm of epicardial coronary arteries has been accepted as an additional functional mechanism of transient angina [30]. Angina is the clinical symptom of myocardial ischemia, which occurs when the cardiac cells do not have adequate oxygen for myocardial oxidation. This occurs as a result of a loss of balance between the oxygen demand of myocytes and the delivery of oxygen to the myocytes [31]. The use of beta-blockers in these patients reduces the consequences of ischemia by decreasing the heart rate, myocardial contractility, and blood pressure. By decreasing the heart rate, the diastolic time is increased, which improves the oxygen supply to the myocytes by increasing coronary filling time. Reducing myocardial contractility and blood pressure results in reduced oxygen demand for the heart [29].

Beta-blockers are also considered first-line therapy for controlling the ventricular rate in patients with atrial fibrillation, though only in patients without reduced ejection fractions. In patients with a reduced ejection fraction, they are not that beneficial [32]. They act by decreasing the rate of conduction through the atrioventricular (AV) node and are preferred to digoxin or calcium channel blockers in patients with a history of myocardial infarction or heart failure [22]. In the setting of acute atrial fibrillation, intravenous administration of various beta-blockers such as esmolol, propranolol, and metoprolol has yielded positive results; similarly, in the case of chronic atrial fibrillation, the administration of oral drugs such as atenolol, bisoprolol, metoprolol, nadolol, and propranolol has been shown to be effective in ventricular rate control [32].

Unlike the above-mentioned indications for the usage of beta-blockers, the use of beta-blockers in hypertension without compelling indications is controversial. Current evidence suggests that initiating treatment of hypertension with beta-blockers leads to a modest decrease in CVD with little to no effect on mortality [20].

Non-cardiovascular Indications of Beta-Blockers

Propranolol is often used to mask the physical manifestation of performance anxiety. It relieves physical symptoms such as increased heart rate, respiration rate, change in blood pressure, and skin conductance [17].

Migraine is one of the most common neurological problems in primary care. According to the global burden disease study, migraine is second among the global causes of disability [33]. Beta-blockers are frequently used for the prophylactic management of migraine and are about 50% effective in producing a reduction of >50% in the frequency of new attacks [34].

Hyperthyroidism is a pathological condition in which excess thyroid hormone is synthesized and secreted by the thyroid gland. The most common cause of hyperthyroidism in parts of the world with sufficient iodine in their diet is Grave’s disease. Hyperthyroidism often presents with symptoms such as palpitations, heat intolerance, weight loss, and tremors. Propranolol can be used to treat the peripheral symptoms of thyroid hormone excess and, additionally, slightly decrease the conversion of thyroxine (T4) to triiodothyronine (T3) [35].

Pheochromocytoma and paraganglioma are rare neuroendocrine tumors that are manifested by a classic triad of headache, palpitation, and profuse sweating. They are characterized by the excessive secretion of catecholamines [36]. For these conditions, beta-blockers are often co-administered to control the tachycardia before the surgery, but this is to be done only after the administration of alpha-blockers. The use of beta-blockers without alpha-receptor blockade may result in hypertensive crises due to unopposed alpha-receptor stimulation [37].

Adverse cardiac effects due to beta-blockade

Beta-blockers demonstrate a dose-dependent effect on intrinsic sympathomimetic activity [38]. This mechanism is pivotal in the adverse cardiac effects caused by beta-blockers. Another concern regarding the use of beta-blockers in patients with HF is the occurrence of other symptoms, including dizziness due to hypotension and bradycardia. One study that reviewed data from HF trials reported between 1966 and 2002 found that, although beta-blockers were associated with these side effects, the absolute increase in symptoms was small and did not necessitate withdrawal of drug therapy [39]. Slowing of the resting heart rate and the development of sinus bradycardia are normal responses to treatment with a beta-blocker. This effect is less prominent with drugs with intrinsic sympathomimetic activity (ISA).

Nevertheless, all beta-blockers are relatively contraindicated in patients with symptomatic bradycardia that may be associated with sinus node dysfunction, especially if there is a further reduction in rate unless an artificial pacemaker is present. Beta-blockers also depress conduction through the AV node, potentially causing a heart block. Use of a beta-blocking drug can therefore lead to serious bradyarrhythmia in patients with an underlying complete or partial AV conduction defect (i.e., second or third-degree AV block), especially if the patient is also receiving another agent that impairs AV nodal conduction, such as digoxin or a calcium channel blocker. Compounds with ISA may cause less impairment of AV conduction [40].

Contraindications to beta-blocker use

Beta-blockers have been known to be contraindicated in obstructive lung diseases such as chronic obstructive pulmonary disease (COPD) because they can potentially lead to fatal bronchospasm in these patients. Cardio-selective beta-blockers are safe in patients with obstructive lung diseases [41] due to the isolation of the beta-blockade effect on solely the heart, not affecting the lungs. Patients suffering from bronchial asthma should also avoid the use of non-cardioselective beta-blockers. Beta-blockers should also not be administered to patients with heart failure who have comorbidities such as bradycardia, heart block, and hemodynamic instability.

## Review

Guidelines and recommendations for beta-blocker use in pregnancy

The association between beta-blocker exposure in early pregnancy and the risk of congenital malformations is controversial. Beta-blockers cross the placenta [42], and at least some studies in animal models suggest a potential teratogenic effect [43]. A recent systematic review and meta-analysis of studies involving pregnant women by Yakoob et al. [44] did not find an increased risk for congenital malformations overall but did report a significantly increased risk for cardiac defects, cleft lip or palate, and neural tube defects [45].

In general, beta-blockers that are considered to pose less risk to a breastfeeding infant have or are predicted to have, lower levels in breast milk (due to a high degree of plasma protein binding, low lipid solubility, and a short half-life) and relatively low renal excretion. The risks of currently available beta blockers vary widely due to these features [46]. Propranolol is considered the beta-blocker of choice in breastfeeding. Metoprolol is also considered to pose a low risk. Acebutolol, atenolol, and nadolol are favored least because of their relatively high milk levels and possible side effects in breastfed infants [9]. Beta-blocker use is associated with hypoglycemia since beta-blockade inhibits glycogenolysis caused by activation of the sympathetic nervous system [47]. The question arises whether this also holds true for neonates after maternal use. Hypoglycemia, and especially prolonged hypoglycemia, can cause severe brain injury in neonates [48,49]. Therefore, it is important to know whether exposure to beta-blockers in utero or through lactation substantially increases the risk for bradycardia and hypoglycemia in neonates and to what extent [50].

Overview of the Effects of Pregnancy on Cardiovascular Function

Pregnancy is a dynamic stage of cardiovascular change for the mother. There are significant physiological changes that occur during pregnancy, such as anatomical, hematological, renal, metabolic, respiratory, and endocrine changes that affect the maternal cardiovascular system. During pregnancy, the maternal heart experiences structural and functional adaptations. These changes are necessary for the pregnant mother to undergo in order to healthily maintain and nurture the fetus with adequate oxygen and nutrients and to ensure a successful delivery [51,52]. Cardiovascular changes start occurring early on in pregnancy, by the sixth week. Cardiovascular changes include increases in cardiac output, heart rate, arterial compliance, extracellular fluid volume, and decreases in blood pressure and total peripheral resistance. Cardiac output (CO) increases by 50% during pregnancy due to the increased metabolic needs of tissues and organs that require increased blood flow. Thus, increases in cardiac output are associated with increases in stroke volume and heart rate [52-54]. Maximum cardiac output peaks at 16 to 20 weeks of gestation, then plateaus after mid-pregnancy with a minimal fall at term [53]. The probable event that incites increased cardiac output is peripheral vasodilation. Vasodilation is mediated by endothelium-dependent factors such as nitric oxide, upregulated estradiol, and prostaglandins (PGI2). This peripheral vasodilation leads to a fall in systemic vascular resistance (25%-30%), and to compensate for this, cardiac output increases during pregnancy [51,53]. An increase in stroke volume is seen during pregnancy due to an early increase in ventricular wall muscle mass and end-diastolic volume. Preload is higher due to an increase in venous return and larger left atrial volumes during the first through third trimesters. The increase in volume load triggers a cardiac restructuring response that mainly consists of left ventricular geometric changes and spherical dilatation. The response to this cardiac remodeling is to maintain an adequate volume of circulating blood. This remodeling, which peaks in the second trimester, in turn, increases myocardial contractility [51,55,56]. These cardiovascular changes assess maternal cardiac function.

It is noted that systemic blood pressure falls during the first and second trimesters due to reduced vascular tone and decreased systemic vascular resistance. The decrease in blood pressure is characterized by both decreases in systolic and diastolic blood pressure. However, in the third trimester, blood pressure begins to rise to pre-gestational levels before term [51,53,54]. Although brachial blood pressure is the more commonly measured cardiovascular parameter in pregnancy, central blood pressure is much more helpful in diagnosing abnormal cardiovascular pathophysiology than brachial blood pressure. This is because central blood pressure is affected by arterial stiffness and pulse wave reflection in the arterial tree. Interestingly, central blood pressure and arterial stiffness have been found to be higher in the preclinical and clinical stages of preeclampsia. Other relevant findings are that diastolic dysfunction and impaired myocardial relaxation were evident in 17.9% and 28.4% of normal-term pregnancies, respectively. These cardiac findings are similar to those in preeclampsia [55-57]. The above cardiovascular changes can be easily misinterpreted as pathological findings if unfamiliar with the pregnancy process, but they can also assist in monitoring and screening for future complications during pregnancy and postpartum. The mechanisms that are responsible for the hemodynamic changes during pregnancy are still not fully understood and deserve further investigation in order to effectively manage any maternal cardiovascular complications, such as preeclampsia, that can arise from maternal cardiovascular maladaptation in both normal and abnormal pregnancies [55,57]. 

Current Evidence for Beta-Blocker Use in Pregnancy

Beta-blockers are one of the most commonly used classes of antihypertensives for treating cardiac conditions and chronic hypertension in pregnant women. Chronic hypertension is becoming increasingly prevalent in pregnancy due to higher obesity rates in women of reproductive age and increasing maternal age [45]. Exposure to antihypertensive medications in early pregnancy is not uncommon. Hypertension in pregnant women remains a major cause of maternal and fetal morbidity and mortality that is complicated in 10% of pregnancies [58]. The hypertensive disorders of pregnancy include pre-existing and gestational hypertension, preeclampsia, and eclampsia. Treatment agents and therapy goals for hypertensive disorders of pregnancy have long been debated and remain controversial [59]. The goal of antihypertensive treatment in the management of hypertension in pregnancy is to lower blood pressure in order to prevent complications due to hypertension during the course of pregnancy [58,59]. In pregnant women with hypertension, target blood pressure is recommended to be >140-150 mmHg for systolic and >90-100 mmHg for diastolic blood pressure [60].

Beta-blockers are one of the most used antihypertensives in the US, UK, Canada, and Europe. According to professional organizations and societies (i.e., the American College of Obstetricians and Gynecologists (ACOG)), recommended first-line therapy treatments for hypertension (non-severe) in pregnancy are beta-blockers together with calcium-channel blockers and methyldopa. The most commonly used agents are labetalol (a beta-blocker), nifedipine (a calcium channel blocker), and methyldopa (an alpha-2 adrenergic agonist) [45,58-61]. However, in severe hypertension, the treatment recommendations are intravenous hydralazine, intravenous labetalol, and calcium-channel blockers (in particular, short-acting oral nifedipine), and the gold standard treatment for preeclampsia continues to be magnesium sulfate [59,60].

Exposure to beta-blockers during pregnancy has been associated with intrauterine fetal growth restriction, preterm birth, and perinatal mortality. Labetalol, which is a beta-blocker with alpha-1 blockade, has not been associated with the adverse neonatal outcomes that other beta-blockers are associated with [61]. Hence, labetalol is considered safe for use during pregnancy. Labetalol is a lipophilic drug that easily crosses the placenta and is readily absorbed, with peak plasma concentrations within 60-90 minutes. In studies done on an in vivo placental transfer of labetalol, fetal/maternal serum ratios were about 0.5 with an unexplained outlier of one [61,62]. It has been shown that clearance (CL) of labetalol (also nifedipine) after oral administration appears to be higher in pregnant women versus non-pregnant individuals. A pharmacokinetic study predicted that labetalol clearance and bioavailability increase approximately 1.4-1.6 fold during pregnancy and concluded that these increases were most likely mediated by increases in maternal hepatic bilirubin UDP-glucuronosyltransferase 1A1 (UGT1A1)-mediated intrinsic clearance. Pregnant women were also shown to have enhanced first-pass metabolism. According to these findings, it is necessary that higher labetalol (also nifedipine) doses be given and/or more frequent dosing may be needed during pregnancy to maintain desired concentrations and prevent therapeutic failure [62,63]. There is also an association found between gestational age and lean body weight on the impact of the pharmacokinetics of labetalol. The dosing should be adjusted according to lean body weight rather than total body weight to reduce excessive drug exposure to both mother and fetus. An increase in gestational age correlates to an increase in the clearance/bioavailability ratio, which requires dosage adjustments as the pregnancy prolongs until term [63]. Labetalol has proven to be one of the safest beta-blockers to be used during pregnancy, albeit with some potential risks and precautions.

Safety Concerns and Potential Risks of Beta-Blocker Use in Pregnancy

Beta-blockers come in different classes with different physiologic and pharmacologic properties (i.e., more beta-1 selectivity versus beta-2 selectivity). Other beta-blockers, such as labetalol and carvedilol, have alpha-1 blocking selectivity that is associated with vasodilation [64]. It is imperative to differentiate between the classes of beta-blockers when studying clinical outcomes. The challenge of beta-blocker usage in pregnancy occurs when treating not only hypertensive disorders but also cardiac conditions (i.e., arrhythmias, valvular heart disease, congenital heart disease, etc.) in pregnant women. Both beta-adrenergic blockers and alpha/beta-adrenergic blockers are very often used to control arrhythmias and ventricular dysfunction in pregnant women with cardiovascular disease [64,65]. Since both hypertensive and cardiac conditions are treated with beta-blockers, it is crucial to assess maternal and fetal risks to ensure the safety of the medication.

There has been conflicting and contradictory evidence on the use of beta-blockers during pregnancy due to potential adverse outcomes and scarce evidence of a safety profile. Beta-blocker usage during pregnancy has been associated with preterm birth, small for gestational age (SGA) neonates, fetal cardiac malformations, perinatal mortality, and other teratogenic effects [45,64-68]. Precautions must be taken when using beta-blockers, specifically labetalol, particularly in pregnant patients with cardiopulmonary conditions, as this will make labetalol contraindicated [69]. Because beta-blockers can cross the placenta, it has been suggested that there are potential teratogenic effects. Previous studies did not find an increased risk for congenital malformations, but an increased risk for cardiac defects, cleft lip or palate, and neural tube defects was reported. However, it has been suggested that underlying maternal hypertension may be a risk factor for malformations [45,65,66]. Exposure to any kind of beta-blocker class, including labetalol, during pregnancy, was found to be associated with SGA neonates. In pregnant women with heart disease, it was found that high doses of beta-blockers resulted in a five-fold increased risk of an SGA infant and a two-fold increased risk among those treated with a low dose, showing an apparent dose-response relationship [66,68]. In another study, exposure to atenolol and labetalol during pregnancy was associated with increased odds of SGA infants. In the same study, exposure to metoprolol and propranolol during pregnancy did not show an association with SGA infants. It is suggested that it may be due to an effect associated with specific medications within the class of certain beta-blockers [67]. Fetal growth restriction was found to be an adverse effect of specific beta-blocker exposure in pregnant women with cardiovascular disease, whereas, with alpha/beta-adrenergic blockers (carvedilol, labetalol), there was no association of fetal growth restriction [65]. In comparing the efficacy of both beta-blockers and calcium-channel blockers, both antihypertensives were found to be effective for lowering blood pressure in pregnancy. However, evidence suggests that beta-blockers, including labetalol, may lower birth weight. In turn, evidence favors calcium-channel blockers for reducing the risk of preeclampsia and eclampsia with no impact on gestational diabetes or low birth weight risk [70]. Despite conflicting evidence, beta-blockers, specifically labetalol, are still considered a mainstay treatment for hypertensive disorders in pregnancy.

Overview of the Effects of Beta-Blockers on Fetal and Neonatal Development

The effects of beta-blockers on fetuses and neonates have been reported to include intrauterine growth restriction (IUGR), SGA, fetal hypoglycemia, and fetal bradycardia. Intrauterine growth restriction and SGA are some of the most widely reported adverse outcomes from the use of beta-blockers in pregnancy. A single-center retrospective cohort at Oslo University Hospital surveyed 540 pregnancies to identify pregnant women treated with beta-blockers such as metoprolol and compare them to controls. The authors of this study found nearly twice the incidence of fetal growth restriction (FGR) in this cohort and further calculated a five-fold risk increase of FGR in infants whose mothers were being treated with high-dose beta-blockers. In the case of low-dose beta-blockers, the risk was observed to be increased two-fold [68]. Another retrospective cohort identified 175 pregnant women with heart disease and matched them with a group of 627 pregnant women from the overall nearby population in Denmark. The cohort concluded a nearly two-fold increase in the incidence of SGA (29.4% vs. 15.3%, p<0.05) in pregnant mothers with heart disease treated by beta-blockers [71]. Data from 60 hospitals in 28 countries on pregnant women taking beta-blockers were combined in a retrospective study. The results showed a lower adjusted birth weight of 100g (3140g vs. 3240 g, p=0.002) [72]. Mendelian randomization estimates have been used to postulate that beta-blockers may negatively impact birth weight [70].

To examine if different subtypes of beta-blockers carry different levels of associated risk of IUGR, a single-center retrospective study looked at 378,238 pregnancies over the span of a decade and identified 4,847 pregnancies with beta-blocker exposure. The study found an increased risk of SGA with atenolol and labetalol, with odds ratios (OR) of 2.4 and 2.9, respectively [67]. Mothers taking propranolol and atenolol were found to have a doubled incidence of FGR in a similar single-center retrospective study. The study details a hypothesis that beta-blockers cause increased vascular resistance in the pregnant mother and their fetus, caused by hemodynamic changes leading to decreased uteroplacental blood flow and increased resistance to blood flow in the placenta and myometrium [65]. One study examined the effect of propranolol on fetal and maternal heart rates and their variability in mice. Propranolol was found to significantly decrease fetal heart rate and increase fetal heart rate variability [73]. One retrospective cohort grouped 158 pregnancies into three groups: those treated with carvedilol (alpha/beta-adrenergic), a beta-adrenergic blocker, and controls. This cohort, performed at a single center, examined all groups for the incidence of FGR. Beta-adrenergic blockers were observed to be associated with FGR, with the type of beta-blocker causing a varied incidence. Carvedilol had no association with FGR [65].

Exposure to beta-blockers during pregnancy has an increased risk for neonatal hypoglycemia. Two million pregnancies were surveyed in a cohort performed in 2016. Of those two million, around 10,585 pregnancies were exposed to beta-blockers at the time of delivery. In this cohort, it was found that beta-blocker exposure was associated with neonatal hypoglycemia and bradycardia, with adjusted odds ratios of 1.58 and 1.29, respectively [74]. A study of 584 full-term infants with beta-blocker exposure matched to 75,000 unexposed mother-infant pairs showed a three-times risk for developing hypoglycemia (relative risk (RR): 3.1). A two-times increased risk of feeding problems (RR: 1.8) was also determined [75]. A single-center study also found a similar preliminary association between beta-blocker exposure and neonatal hypoglycemia and recommended neonatal blood glucose monitoring [71]. Beta-blocker exposure has been shown to result in other deleterious cardiovascular outcomes as well. A decrease in pulmonary trunk systolic velocity in fetuses exposed to atenolol during the 28th-40th weeks of gestation was determined in a wide systematic review. This review also surveyed the effects of beta-2-agonists, corticosteroids, non-steroidal anti-inflammatory drugs (NSAIDs), metformin, insulin, highly active antiretroviral therapy (HAART), and chemotherapy on fetal cardiac function [76].

Untreated hypertension in pregnancy carries a high risk of stroke and fetal adverse outcomes. Labetalol is regarded as the recommended beta-blocker of choice for hypertension in pregnancy. To find out whether it is suitable or not, many randomized controlled trials have been performed. These were surveyed through multiple databases in a systematic review to find that no differences in adverse maternal or fetal outcomes were found when compared to oral or sublingual nifedipine or parenteral hydralazine [75]. A prospective cohort performed in a single center in Cape Town, South Africa, over seven years observed no significant association with beta-blocker usage and decreased fetal birth weight among certain subtypes of structural heart diseases [77]. However, a retrospective cohort in the city of Saskatchewan, Canada, found a higher incidence of SGA (adjusted odds ratio (AOR) of 1.95) and neonatal hospitalization rates (AOR of 2.17) in pregnant hypertensive mothers taking labetalol versus methyldopa [78]. There are instances when the use of beta-blockers has been shown to greatly benefit fetus survival. A case of a fetus in the third trimester diagnosed with hypertrophic cardiomyopathy (caused by a first-degree familial beta-myosin heavy chain mutation) by echocardiogram showed diastolic dysfunction and septal hypertrophy. Maternal propranolol was administered in the third trimester and continued after birth to the neonate. By nine months, the infant had shown evidence of regression of septal hypertrophy [79].

Inherited long QT syndrome (iLQTS) presents a higher risk of IUGR and preterm delivery, along with a higher maternal risk of arrhythmia. It may be diagnosed prenatally with a family history, a characteristic arrhythmia, bradycardia, and associated findings such as AV block or torsades de pointes (TdP). One case of iLQTS was reported to be treated with trans-maternal nadolol, which was shown to be effective in the restoration of fetal sinus rhythm [80]. A review examining beta-blocker usage in pregnant women with iLQTS observed the subsequent effects on the uterus, fetal birth weight, and fetal glycemic levels. Beta-1 selective receptor blockers such as metoprolol and bisoprolol were found to be associated with a lower incidence of adverse outcomes and are preferred (excluding atenolol) for first-line therapy in these pregnant women. The authors of the review insist that multidisciplinary care must be taken to prevent adverse outcomes [81]. Beta-blockers may have a cardio-protective mechanism, as postulated by a study exploring beta-blockers as a strategy in the treatment of preeclampsia. The study examines the effects of beta-blockers such as carvedilol, bisoprolol, and metoprolol on endothelial secretion and the expression of anti-angiogenic and pro-angiogenic factors. Beta-blockers were found to offset the endothelial dysfunction seen in in vitro preeclampsia models by suppressing the secretion of anti-angiogenic factors and increasing the expression of pro-angiogenic factors [82].

Effect of beta-blockers on lactation

Beta-blockers are used to treat multiple diseases in lactating women. They can pass into breast milk and harmfully affect the neonate, causing hypoglycemia and bradycardia [50]. Beta-blockers inhibit beta-1 and beta-2 receptors. The blockade leads to a decrease in heart rate and heart contractility. This decrease in cardiac output lowers blood pressure. Cardiac output is mostly dependent on heart rate in newborns. Thus, beta-blockers can cause reduced organ perfusion. Hypoglycemia occurs from a blockade of glycogenolysis caused by decreased sympathetic nervous system activity [50]. Long-term hypoglycemia can lead to severe brain injury [48]. Exposed infants can also develop drowsiness, lethargy, poor feeding, and weight gain [83].

Principles determining drug safety in breastfeeding include factors affecting milk-drug concentration that are drug-related and maternal-related. Drug-related factors are dosage, bioavailability, lipid solubility, protein binding, molecular weight, pKa, half-life, active metabolites, and mechanism of elimination. Maternal-related factors are maternal dose, pharmacogenomics, renal, and hepatic function. Infant health needs to be considered simultaneously while prescribing these drugs. An unwell infant’s health is at risk of deterioration if exposed to such medicines. Premature infants are also at a higher risk of drug accumulation due to decreased clearance capacity [83]. Breast milk drug concentration depends on the pharmacokinetics of each individual beta-blocker. Labetalol and propranolol are in low concentration in breast milk; hence, they are prescribed widely to lactating women [84]. Whereas atenolol and sotalol are in high concentrations in breast milk, leading to adverse effects [50,85]. Propranolol and metoprolol are also preferably used during lactation due to available evidence supporting their clinical efficacy and safety and their active use in the pediatric population [83].

Metoprolol is safe to use during breastfeeding with no reported adverse effects. It has low bioavailability, a short half-life, and is only found in small amounts in breast milk (0.5%-2% of the maternal weight-adjusted dose). It can be used in infants from one month onwards. Metoprolol succinate is the drug of choice if it is available. Bisoprolol has an unfavorable profile. It has low protein binding, good oral bioavailability, a longer half-life, moderate lipid solubility, and high renal excretion, which can lead to its accumulation in an infant. It can be used with caution. Carvidilol has a better profile with very high protein binding, low oral bioavailability, and a shorter half-life, but has high lipid solubility and passes into breast milk readily. It is the second choice if metoprolol succinate cannot be used. It should still be used with caution. Nebivolol has an unfavorable profile, especially in poor metabolizers with a 23x higher plasma concentration. It has wide variability in peak plasma concentration and half-lives in each patient. It also has high protein binding and high lipid solubility. Drug passage into breast milk is expected, but it is difficult to measure the amount and the infant’s response. Nebivolol should not be considered unless other beta-blockers cannot be used and should be used with extreme caution [83].

Evidence regarding infant exposure to drugs during lactation is scarce. This leads to poor decision-making, and the mother is eventually advised to stop breastfeeding. Breastfeeding should not be ruled out completely, considering its benefits. An informed decision can be made with consideration of drug properties, adverse effects, pediatric use, and pharmacokinetics. Drugs should be used at the lowest effective dose and for the shortest duration to decrease exposure to the infant [83].

Use of beta-blockers in certain pediatric conditions

Infantile hemangiomas are the most common childhood vascular tumors. A review of a hospital's experience with propranolol use in managing infantile hemangioma was undertaken, in which the majority of the participants had pre-existing conditions like preterm birth and infantile hemangioma with predominant involvement of the orbits and periorbital areas. Propranolol was started at an age of 7.7m +/- 10.5 weeks of age, and nearly half of the participants were positively impacted by the use of propranolol, with a decrease in the size of the tumor and a reduced risk of complications [86]. Also, when propranolol was given at a dose of 3 mg/kg per day, twice daily, it showed significant improvement towards the end of the course. However, side effects like headaches and dizziness were also reported by the majority of people [87].

Marfan syndrome is a genetic defect affecting connective tissues, leading to easy damage to the fibers that support blood vessels and tissues like the eyes, heart, and skeleton, resulting in aortic aneurysm, aortic dissection, and skeletal deformities like pectus excavatum and pectus carinatum. Beta-blockers are commonly utilized in the treatment of Marfan syndrome. These drugs operate by blocking the impact of stress hormones like adrenaline on heart contractions and lowering blood pressure by reducing the stress on the aorta. Beta-blockers can help slow down aortic root dilation progression and decrease the risk of aortic dissection. Individuals with evidence of aortic root dilation due to Marfan syndrome are usually treated with beta-blockers starting in their childhood or adolescence, which continues through adulthood. It is critical to keep in mind that while beta blockers cannot cure Marfan syndrome, they can help manage its symptoms and decrease the risk of life-threatening complications. The study conducted by Alex Pitcher et al. [88] aimed to compare the effects of angiotensin II receptor blockers (ARBs) versus control and ARBs versus beta-blockers in individuals with Marfan syndrome who had not undergone aortic surgery. Most of the participants were in the younger age group. The main outcomes indicated that Marfan syndrome patients who were not treated with aortic surgery experienced a considerable reduction in the rate of aortic root Z-score growth, but when used with either ARBs or beta-blockers, the growth rate of aorta dilation was halved.

Future directions and developments for beta-blockers

Beta-blockers include a plethora of molecules with vastly different pharmacokinetic and pharmacodynamic characteristics, with clinical indications stemming from large randomized clinical trials. There are new indications still emerging. In this section, we will assess their use in cardiovascular medicine, focusing on hypertension, in which beta-blockers are highly utilized, and discuss the evidence for potential additional therapeutic cardiovascular effects [89].

Beta-blockers remain under-prescribed in populations at increased risk for cardiovascular disease because of tolerability and safety concerns. Among the newer agents, labetalol, carvedilol, and nebivolol have been approved for use in the United States. Nebivolol possesses both beta1-selectivity and nitric oxide-mediated vasodilatory effects, while carvedilol has attractive effects on insulin resistance and exhibits antioxidant effects. Newer beta-blockers may overcome concerns about efficacy, adverse effects, and tolerability while delivering cardiovascular protection [90].

Potential New Indications for Beta-Blockers in Pregnancy

Over the past two decades in the United States, there has been an increase noted in both maternal mortality and the incidence of arrhythmias in pregnancy. The state of pregnancy poses a risk of arrhythmias, and the risk is even greater in women with pre-existing arrhythmias that can reoccur during pregnancy [91]. Beta-blocker therapy has been the gold standard for treating hypertensive disorders in pregnancy but can also be used to treat tachyarrhythmias effectively and safely. In particular, propranolol and metoprolol are preferred to treat tachyarrhythmias in pregnancy due to the fact that there is no risk of adverse effects on fetal health and they are safe to use during lactation [45,91]. Atenolol, on the other hand, has shown a greater risk of fetal adverse effects and is not recommended during lactation [45]. Certainly, further studies involving pregnant women with arrhythmias treated with beta-blockers would be beneficial to monitor each stage during gestation, labor and delivery, and post-term for successful maternal and fetal outcomes.

Potential New Indications for Beta-Blockers in Fetal and Neonatal Health 

Beta-blockers cross the placenta and potentially cause physiological changes in the fetus. Beta-blocker exposure has been shown to cause bradycardia and hypoglycemia in neonates. A recent meta-analysis reported an association between beta-blocker exposure and fetal congenital cardiovascular defects, raising concern regarding potential teratogenic effects [67].

The emerging use of topical beta-blockers for treating infantile hemangiomas has changed from using oral corticosteroids since 2008. Clinicians are using timolol maleate gel as the preferred treatment, with good results [92]. The only known adverse effects reported from using topical timolol in neonates are pruritus and sleep disturbance [92]. Compared to using oral propranolol, which can cause hypoglycemia, topical timolol has a safer outcome for neonates. However, standardization of dosing, duration of treatment, and safety profile would be further beneficial in future research for treating infantile hemangiomas.

Heart failure is becoming an increasingly common and significant problem in the field of pediatric cardiology. The most recent and important breakthrough in the pharmacologic treatment of heart failure has been the particular role of beta-blocker therapy. The advent of beta-adrenergic receptor blockade (beta-blocker) therapy, which has shown significant improvements in symptoms, survival, left ventricular function, and reverse remodeling in adults with heart failure, has led to early optimism in its utility for the management of pediatric heart failure [93]. The current agents used and approved for heart failure fall into two major categories: second-generation ‘selective’ and third-generation ‘non-selective’ beta-blockers [94,95]. The second-generation beta-blockers are designed to be preferentially beta-receptor selective. The agents in increasing order of beta-1 selectivity are metoprolol tartrate, metoprolol succinate, bisoprolol, and nebivolol. At high doses, these agents lose their beta-selectivity properties, and their potential benefits may be altered [95,96]. Several of the newer agents have been shown to have additional properties, including anti-oxidant effects, as observed with nebivolol and bisoprolol, and vasodilator effects, as observed with nebivolol. Currently, metoprolol succinate and bisoprolol are approved for heart failure management in adults, with only metoprolol usage being reported and investigated in pediatrics [93].

**Figure 1:**
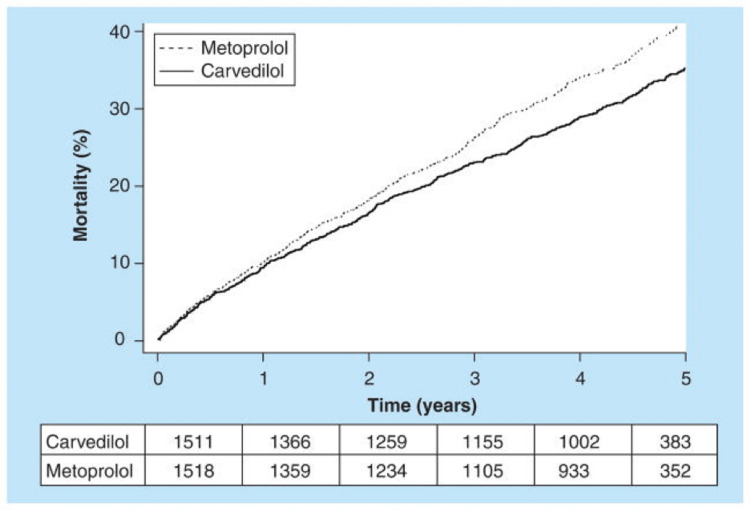
All-cause mortality from the Carvedilol or Metoprolol European Trial (COMET) Reproduced under the terms of the Creative Commons Attribution license. Adult patients with chronic heart failure demonstrated all-cause mortality of 34% (512 of 1511) with carvedilol and 40% (600 of 1518) with metoprolol (hazard ratio: 0.83; 95% confidence interval (CI): 0.74–0.93; p = 0.0017). A total of 3029 patients with heart failure (NYHA II–IV), a prior admission for cardiovascular reasons, and a left ventricular ejection fraction of less than 35% were randomized to metoprolol tartrate or carvedilol and showed a reduction in all-cause mortality in the carvedilol group: 34% (512 or 1511) for carvedilol and 40% (600 of 1518) for metoprolol [102].

The third-generation beta-blockers are non-selective agents that attempt to counteract the adrenergic system at multiple sites. Both carvedilol and bucindolol have beta-1, beta-2, and alpha-1 blockades. Antioxidant, antiapoptotic, vasodilatory, and myocyte energy utilization effects with carvedilol have also been proven [96-99]. Bucindolol lacks these additional properties except for vasodilatation but also has intrinsic sympathomimetic activity, which may ultimately limit its efficacy [100]. The limited impact of bucindolol can also be connected to race after the Beta-blocker Evaluation of Survival Trial (BEST), which demonstrated a lack of survival benefit in black patients compared with non-Black patients, raising the question of whether genetic variations in these subpopulations led to disparate findings [101]. To date, the only head-to-head comparison amongst these agents has been the comparison of carvedilol to metoprolol tartrate, which demonstrated carvedilol to be superior, causing an additional reduction in all-cause mortality by 6% and an increase in median survival by 1.4 years based on extrapolation as shown in Figure 1 [102].

Potential New Indications for Beta-Blockers in the Pediatric Population

In the pediatric population, beta-blockers are FDA-approved for a few indications, such as hypertension and hemangioma. However, beta-blockers are becoming commonly used to treat other indications off-label in the pediatric population without sufficient clinical evidence for safety and efficacy [103]. The literature on beta-blockers in pediatrics follows some case reports that address treating arrhythmias, syncope, hypertrophic cardiomyopathy, and portal hypertension. Due to a lack of comprehensive pediatric studies, dosing, efficacy, and safety have been mainly extrapolated from adult data [104]. A popular beta-blocker of choice to use off-label in pediatrics is propranolol. Propranolol has been used to treat a variety of pediatric cardiac conditions, such as cyanotic spells due to tetralogy of Fallot, long QT syndrome, right ventricular failure, balloon angioplasty of aortic coarctation, and supraventricular tachycardia. Propranolol has also been used to treat non-cardiac conditions such as thyrotoxicosis, Marfan syndrome, migraine prophylaxis, and anxiety disorders [103,104]. With such a large literature gap, there is a need to conduct further studies on treating the pediatric population with beta-blockers for various indications. Pediatrically controlled drug studies that establish the safety and efficacy of beta-blockers are necessary before obtaining FDA approval.

Limitations of existing studies and literature gaps

There is still a knowledge gap in regard to fully understanding the physiological mechanisms and changes during pregnancy. Most of the literature describes cardiovascular changes in pregnancy as a compensation method for volume overload and adaptations that result in cardiac dysfunction until postpartum. Those changes may seem pathological but are in fact normal in pregnancy until complications arise. Further investigation is deserved into the relevance of maternal cardiovascular dysfunction to the development of cardiovascular pregnancy complications, such as preeclampsia. On the pharmacological side of beta-blockers, additional studies are needed to assess and precisely quantify the gestational changes in antihypertensive drug pharmacokinetics during pregnancy and to further elucidate the underlying mechanisms of gestational changes in pharmacokinetics. Also, clinical trials are needed to optimize beta-blocker dose-finding and safety for different gestational ages (first, second, and third trimesters), as well as increased pharmacovigilance during and after pregnancy.

More studies are needed to compare various classes of beta-blockers and other antihypertensive classes of medication in order to create better target therapy strategies for treating the various hypertensive disorders in pregnancy, including prophylactic treatment of preeclampsia and eclampsia. More rigorous research is needed to address the risk and safety profiles of labetalol and other beta-blockers during pregnancy to further validate side effects and adverse fetal outcomes in women with hypertensive disorders and heart disease.

To understand the safety of a drug, it must be compared to other gold-standard therapies. Fifty fetuses were enrolled in 15 Japanese institutions in a prospective study. The study protocol outline was intended to have three treatment groups with sotalol, digoxin, and flecainide in the treatment of antenatal tachyarrhythmias. Any subsequent fetal, neonatal, or maternal adverse effects were evaluated at one month postpartum and at 18 months and 26 months of age [105]. Prospective controlled studies of this nature would give us further valuable insight into adverse outcomes when compared to other therapeutic methods. Conflicting results are found across studies, of which many are single centers or single geographical locations. This may indicate the presence of confounding factors, such as similar genetics, for participants in a study utilizing a single geographical location. Further randomized controlled trials are recommended to separate the underlying maternal condition from the therapeutic effect of the beta-blocker to understand what is causing the lower birth weight [72]. Controlled studies would help establish a robust association between FGR and beta-blocker exposure.

The incidence of peripartum cardiomyopathy and other cardiovascular conditions surrounding pregnancy has shown an increasing trend in the USA. A study utilizing natural language processing on Twitter surveyed posts about pregnancy and adverse outcomes. The study found a rate of 14.8% for adverse outcomes in pregnant women taking beta-blockers, suggesting that there may be further complementary resources for cohort studies of drug safety in pregnancy [106]. The current American Heart Association (AHA)/ American College of Cardiology (ACC)/ Heart Failure Society of America (HFSA) guidelines for obstetric management of cardiovascular conditions include close monitoring for maternal signs of heart failure or cardiovascular instability, along with fetal monitoring. A third-trimester echocardiogram to screen may be considered [70]. Different methods of study and wider cohorts will further shed light on beta-blocker associations with adverse outcomes.

## Conclusions

The use of beta-blockers for treating common cardiovascular conditions during pregnancy, such as hypertension and arrhythmias, is well established despite the limited knowledge of their safety and efficacy profile for maternal, fetal, neonatal, and pediatric health. This review seeks to synthesize the use of beta-blockers in pregnancy, neonates, and the pediatric population for common indications and non-traditional indications.

There is still a large literature gap in how particular beta-blockers fare in safety, efficacy, pharmacokinetics, bioavailability, and appropriate dosage for women who are pregnant or breastfeeding, neonates, and the pediatric population. The current literature on the risks of beta-blocker exposure during pregnancy is somewhat contradictory since studies have found that congenital malformations, teratogenic effects, and low birth weight have resulted from beta-blocker exposure to the fetus. On the other hand, other studies have not concluded that beta-blocker exposure during pregnancy has such effects. Despite conflicting evidence, labetalol has still proven to be the gold standard beta-blocker to use in pregnancy. Current studies have also noted off-label uses of beta-blockers for non-traditional indications (i.e., migraines, pheochromocytoma, infantile hemangioma, and Marfan’s syndrome).

Further studies are needed to delve into current and future developments for beta-blocker indications as well as deeper research into understanding the pharmacokinetics and pharmacodynamics of beta-blockers in pregnant women and women breastfeeding, neonates, and the pediatric population. Safety and efficacy profiles of particular beta-blocker classes are also necessary for guidance and surveillance of treatment.
